# Case Report: Comprehensive evaluation of ECG phenotypes and genotypes in a family with Brugada syndrome carrying *SCN5A-R376H*

**DOI:** 10.3389/fcvm.2024.1334096

**Published:** 2024-03-15

**Authors:** Ngoc Bao Ly, Yoo Ri Kim, Ki Hong Lee, Namsik Yoon, Hyung Wook Park

**Affiliations:** ^1^ Department of Internal Medicine, Chonnam National University Medical School, Gwangju, Republic of Korea; ^2^ Department of Cardiovascular Medicine, Chonnam National University Hospital, Gwangju, Republic of Korea

**Keywords:** Brugada syndrome, family screening, genetic study, *SCN5A*, sudden cardiac death

## Abstract

**Background:**

Brugada syndrome (BrS) is a channelopathy that can lead to sudden cardiac death in the absence of structural heart disease. Patients with BrS can be asymptomatic or present with symptoms secondary to polymorphic ventricular tachycardia or ventricular fibrillation. Even though BrS can exhibit autosomal dominant inheritance, it is not easy to identify the phenotype and genotype in a family thoroughly.

**Case:**

We report the case of a 20-year-old man with variants in *SCN5A* and *RyR2* genes who was resuscitated from sudden cardiac death during sleep due to a ventricular fibrillation. The patient did not have underlying diseases. The routine laboratory results, imaging study, coronary angiogram, and echocardiogram (ECG) were normal. A type 1 BrS pattern was identified in one resting ECG. Furthermore, prominent J wave accentuation with PR interval prolongation was identified during therapeutic hypothermia. Therefore, we were easily able to diagnose BrS. For secondary prevention, the patient underwent implantable cardioverter defibrillator implantation. Before discharge, a genetic study was performed using next-generation sequencing. Genotyping was performed in the first-degree relatives, and ECG evaluations of almost all maternal and paternal family members were conducted. The proband and his mother showed *SCN5A-R376H* and *RyR2-D4038Y* variants*.* However, his mother did not show the BrS phenotype on an ECG. One maternal aunt and uncle showed BrS phenotypes.

**Conclusion:**

Genetics alone cannotdiagnose BrS. However, genetics could supply evidence or direction for evaluating ECG phenotypes in family groups. This case report shows how family evaluation using ECGs along with a genetic study can be used in BrS diagnosis.

## Introduction

Brugada syndrome (BrS) has a higher prevalence in Asian countries (1). It is characterized by an ECG with coved ST-segment elevation in the right precordial leads V1–V2 in the presence or absence of a sodium-channel-blocking agent (2, 3). Hundreds of variants, most commonly in the gene encoding the cardiac sodium channel, have impacts on BrS (4). *SCN5A,* which was first identified to be linked to BrS by Chen et al. in 1998, encodes the voltage-gated, type V, α subunit of the sodium channel (5). *SCN5A* has been proposed to be the major causative gene of BrS and accounts for approximately 20% of familial cases (6, 7). Recent studies have indicated that patients harboring *SCN5A* variants have worse prognosis (2).

In this case report, we present a typical genetic penetrance of a BrS proband and his family with ECG phenotypes. Variant-specific genetic testing is recommended for family members following identification of the causative variant in a BrS index case (8). We evaluated the genetics of first-degree relatives of the proband and thoroughly reviewed the ECG phenotypes of paternal and maternal family members.

## Case description

**Figure 1:**
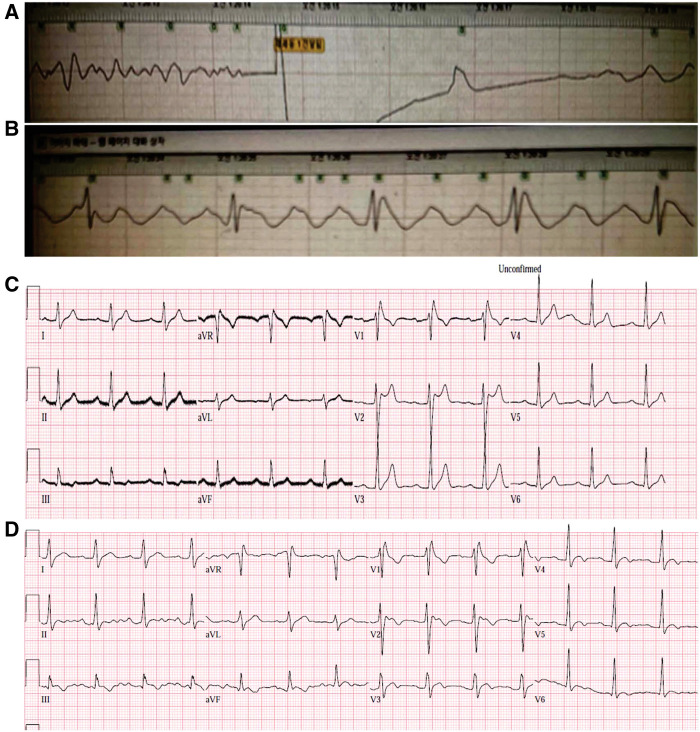
The initial ECG of the index patient, which was recorded on an automated external defibrillator. Defibrillation was performed for the ventricular fibrillation that occurred during transfer of the patient to the emergency room (**A**). After the cardioversion, ST elevation was observed in the defibrillator-patch ECG (**B**). The initial 12-lead ECG showed normal sinus rhythms with first-degree AV block (**C**). The PR interval was 240 ms. A type 2 Brugada pattern ECG was observed in V1 and V2. The V1 and V2 showed dynamic changes that is more like type 1 ECG (**D**). ECG, electrocardiogram.

**Figure 2:**
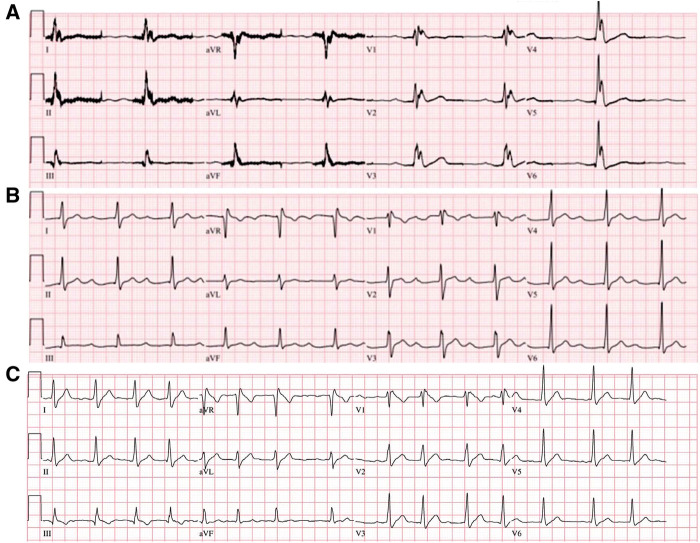
ECG of the index patient during and after therapeutic hypothermia. Widespread J wave accentuation was observed during the hypothermia (**A**). Marked PR-interval prolongation (440 ms) was identified after therapeutic hypothermia (**B**). In a follow-up ECG, a spontaneous type 1 Brugada pattern was identified with an incidental atrial fibrillation (**C**). ECG, electrocardiogram.

**Figure 3:**
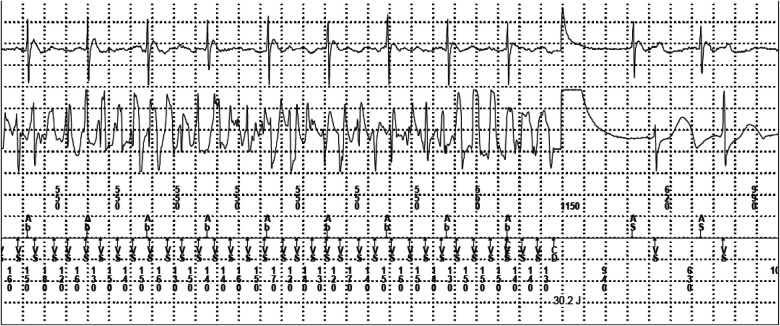
An implantable cardioverter defibrillator shock during sleep was detected and terminated successfully.

A 20-year-old male suffered cardiac arrest immediately after developing chest pain and dyspnea during sleep. Spontaneous circulation was recovered after cardiopulmonary resuscitation. While the patient was being transferred to the emergency room, defibrillation was performed twice due to ventricular fibrillation (Figures 1A,B). The patient did not have underlying diseases or a social history of smoking or alcohol use. Interestingly, the patient's maternal uncle suffered a sudden cardiac death in his 5th decade. On admission, the index patient was in a stable hemodynamic state despite being in a confused mental state and being intubated. Diagnoses were proposed, including acute coronary syndrome and inherited primary arrhythmia syndromes. The initial ECG showed a normal sinus rhythm with a first-degree AV block (PR-interval was 240 ms), and a type 2 Brugada pattern was observed in the V1 lead (Figure 1C). The V1 and V2 showed dynamic changes that is more like type 1 Brugada pattern ECG (Figure 1D) The hematological test revealed increased white blood cells to 13,900 cells/mm^3^. The CRP level was within the normal range, and there were no abnormal results for liver function tests, renal function tests, and coagulation profiles. The serum sodium, potassium, calcium, and magnesium were 142 mEq/L, 3.4 mEq/L, 8.1 mg/dl, and 1.6 mg/dl, respectively. Serum troponin I and T were elevated to 1.823 ng/ml and 0.279 ng/ml, respectively. CK-MB and pro-BNP were within normal ranges (3.59 ng/ml and 28 ng/ml, respectively). The chest radiograph was normal. The brain CT scans and chest CT angiography were nonspecific. Echocardiography revealed no structural heart disease. The coronary angiography showed no significant stenosis. Therapeutic hypothermia management was performed. Prominent J wave accentuation with PR-interval prolongation was identified during the therapeutic hypothermia (Figure 2). In a follow-up ECG, a spontaneous type 1 Brugada pattern was identified with an incidental atrial fibrillation (Figure 2C) The patient was diagnosed with BrS and underwent implantable cardioverter defibrillator (ICD) therapy. After the ICD, the patient suffered ICD shocks due to ventricular fibrillation (VF) during sleep (Figure 3). Therefore, we prescribed quinidine (200 mg twice a day) and cilostazol (100 mg twice a day), which diminished the Brugada pattern on the ECG. After receiving the prescription, VF did not occur again.

**Figure 4:**
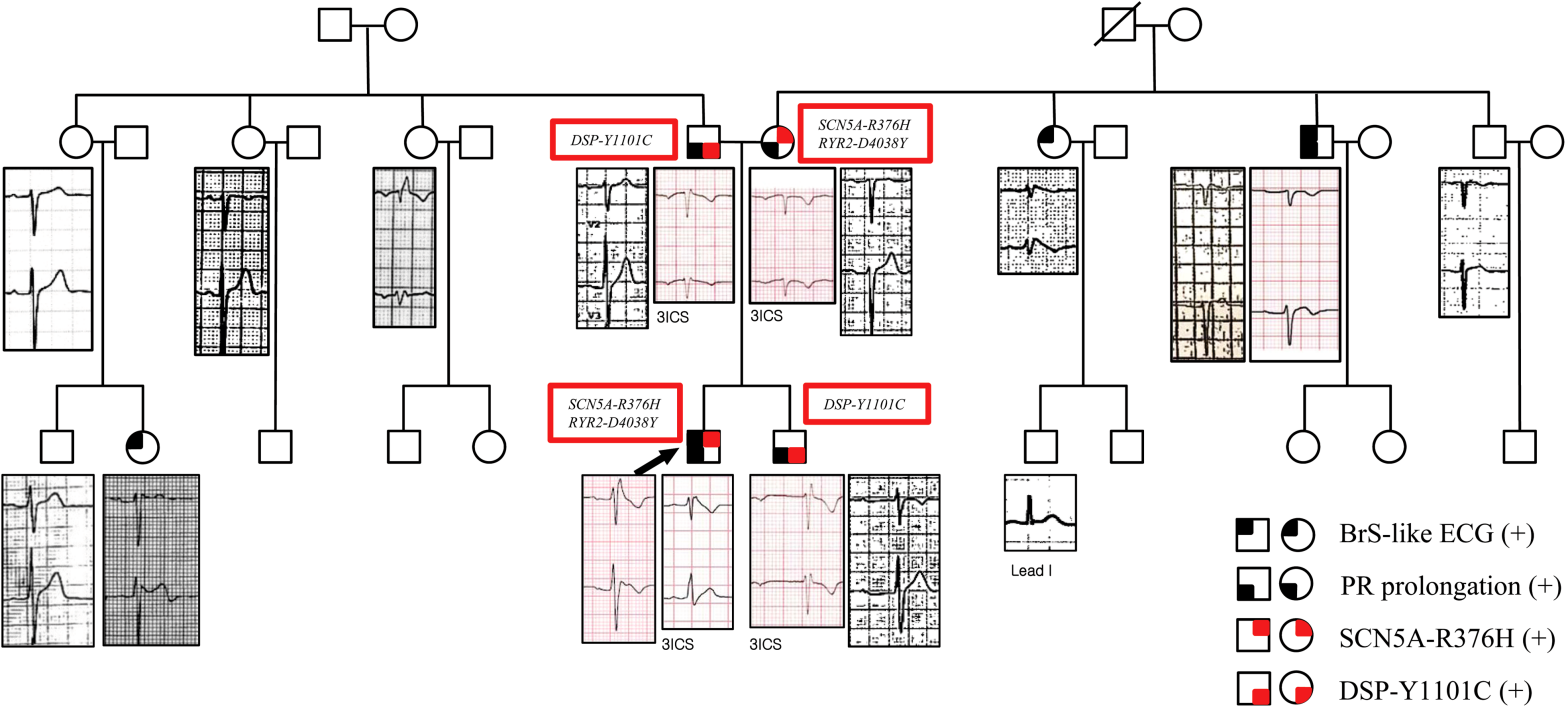
Family pedigree with ECG phenotypes in V1 and V2. The proband's ECG showed a type 1 Brugada pattern in V1. The brother's ECG showed a long PR interval of 480 ms and incomplete RBBB in the 3rd intercostal V1 and V2. The father's ECG showed a long PR interval of 240 ms. The mother's ECG showed a long PR interval of 220 ms without the Brugada pattern on the ECG. One maternal aunt showed a type 1 Brugada pattern in V2. One maternal uncle, who experienced aborted sudden cardiac death, showed a suspicious BrS-pattern on the ECG in V1. One paternal aunt showed an RBBB with a normal PR interval. One paternal female cousin showed a type 2 Brugada pattern with a PR interval of 200 ms in V2. Information about the genetic mutations of first-degree relatives of the patient is also given. The squares represent males, and the circles represent females. The squares/circles with black in the left upper quadrant represent the BrS-like ECG. The squares/circles with black in the left lower quadrant represent PR prolongation. The squares/circles with red in the right upper quadrant represent the SCN5A-R376H variant. The squares/circles with red in the right lower quadrant represent the DSP-Y1101C variant. 3ICS, third intercostal space; ECG, electrocardiogram; BrS, Brugada syndrome.

The proband had a father, a mother, a brother, three paternal aunts, one maternal aunt, and two maternal uncles. We performed genetic tests using next-generation sequencing (NGS) in all first-degree relatives and evaluated the ECGs of almost all family members (Figure 4). All the ECG of the first-relatives were obtained in the usual V1,V2 location and in the 3rd intercostal space to identify the coved pattern ECG. The other family member screening ECGs were obtained in the usual V1, V2 location. The index proband and one maternal aunt had a type 1 Brugada pattern in V2. One maternal uncle, who experienced aborted sudden cardiac death, had a suspicious BrS-pattern on the ECG in V1. One paternal female cousin showed a type 2 Brugada pattern in V2. The other family members did not show Brugada pattern.

The proband and the mother had the *SCN5A-R376H* (*c.1127G>A*) and *RyR2-D4038Y* (*c.12112G>T,* variant of unknown significance) variants*.* They did not show features of catecholaminergic polymorphic ventricular tachycardia. The father and brother had the *DSP-Y1101C* (*c.3302A>G,* variant of unknown significance) variant. They did not show features of arrhythmogenic right ventricular cardiomyopathy. The proband did not have *DSP-Y1101C. SCN5A-R376H* was a likely-pathogenic variant for BrS. All the mutations were heterozygous. Genetic tests were not conducted on the other family members.

## Discussion

BrS could be diagnosed when there was ST-segment elevation with type 1 morphology in one or more precordial leads in the second, third, or fourth intercostal space either spontaneously or after administration of a provocative drug (3, 9). The index patient had a spontaneous type 1 Brugada pattern ECG, unexplained cardiac arrest, documented ventricular fibrillation, aborted sudden cardiac death in a second-degree relative, and a pathogenic variant in a BrS-susceptibility gene. Therefore, this patient had 8 points according to the Shanghai score system, and BrS could be probably or definitely diagnosed.

In this case report, we presented a proband with typical demography and clinical characteristics of BrS. Interestingly, there was one maternal uncle who experienced aborted sudden cardiac death in his 5th decade. Since inheritance was suspected, we decided to evaluate the family pedigree and conduct a genetic study in the patient's first-degree relatives using the NGS technique. Also, we evaluated the ECGs of the proband's other family members.

BrS is an inherited arrhythmogenic disease with incomplete penetrance that is more frequent in those of Asian ethnicity (7). In up to 60% of patients, the disease phenotype can be sporadic (10). Mutations in more than 23 genes have been linked to BrS. The gene most frequently associated with BrS is *SCN5A.* Variants in *SCN5A* have been found in 15%–30% of probands (7, 11). More than 300 *SCN5A* variants have been associated with BrS (12). Currently, the yield of the genetic test, percentage of controls with rare missense variants in major genes, and signal-to-noise ratio of *SCN5A* have been reported as 20%, 2%, and 10:1, respectively (13). The *SCN5A* gene, located at chromosome 3p21 with 28 exons, is a member of the human voltage-gated sodium channel gene family and encodes the α subunit of the main cardiac sodium channel Na­_v_1.5 (14, 15). BrS-related *SCN5A* variants are usually loss-of-function variants. One of the missense mutations of *SCN5A*, known as the *SCN5A*–*R376H* variant, occurs in the first pore segment of the *SCN5A* channel, which has been reported to be associated with highly variable phenotypic manifestations from BrS to conduction disease (16, 17). In the whole cell currents study using patch clamp, *SCN5A*–*R376H* demonstrated a significant current reduction.

The proband had the same variants as his mother; that is, he had variants in the *SCN5A* and *RyR2* genes. *SCN5A-R376H* shows autosomal dominant inheritance; therefore, it is likely the proband inherited the variants from his mother. Since BrS patients' ECGs show very dynamic changes, it is not easy to diagnose BrS with one ECG at one moment in time. Even though sodium channel blockade testing has a variable sensitivity and specificity (18), if an ajmaline provocation test had been performed in the mother, an ECG indicative of BrS may have been revealed. In any case, the mother did not have any ECGs that indicated BrS. Even the same *SCN5A* variant can have different phenotypes. Thus, the phenotype may have individual or family differences or be affected by certain regulatory genes as well as environmental factors (2, 17). Therefore, the genetics of BrS must be considered on a family-by-family basis. The father and brother had long PR intervals and the *DSP-Y1101C* variant. Desmoplakin (*DSP*), the first desmosomal gene linked to autosomal dominant arrhythmogenic right ventricular cardiomyopathy, connects desmosomes and intermediate filaments to maintain the mechanical integrity of cardiomyocytes (19–21). Interestingly, *DSP* variants were identified in some BrS patients without any known BrS-associated variants (21, 22). We presume the loss of *DSP* function could be the delayed depolarization pathogenicity for BrS because of its effects on the reduction of sodium current along with its slow conduction velocity. However, an association between the *DSP* gene and BrS remains controversial (23). Also, this assumption has a big limitation. The *DSP-Y1101C* showed unknown significance in this case. The potential implication with *DSP* and BrS would only be reasonable when functional studies reveal the significance of the variant. If the *DSP-Y1101C* variant is pathogenic or likely pathogenic for BrS, the proband's father and brother could also be at risk of BrS (20). In order to verify our assumption about *DSP-Y1101C* and BrS, functional studies are required absolutely.

It is also interesting that the proband with the *SCN5A-R376H* variant exhibited J wave accentuation and a long PR interval during the hypothermia treatment. Hypothermia can aggravate a BrS-type ECG by causing phase 2 reentry by slowed activation of *I­_Ca_* and leaving *I_to_* unopposed (9, 24). The ECG changes in the proband were expected because the *SCN5A*-*R376H* variant can be associated with BrS and conduction disease.

Although the mother did not show a spontaneous BrS-pattern ECG, one maternal aunt showed a type 1 BrS ECGs and an uncle who experienced aborted sudden cardiac death showed a suspicious BrS-pattern ECGs. Therefore, BrS genetic penetrance was assumed in those maternal family members, even though genetic exams were not conducted on them. BrS genotype and phenotype do not always match (17, 25, 26). Clinical elements including age, sex, or medications may also modify the disease's expression. To investigate the association between the BrS ECG phenotype and genotype of the *SCN5A* variant, Probst et al. studied 13 families with Brugada ECG patterns; ECG phenotypes were only observed in 18% of the *SCN5A* variant carriers and in 61% after drug challenge (27). On the other hand, 8 individuals had Brugada-pattern ECGs but did not carry the familial mutation (27). Clinical manifestations also appear related to hormonal factors (1). Interestingly, clinical presentation has been reported more severe in males than females. In the proband's family survivors of aborted sudden cardiac death were all male.

Risk stratification is the most important and still unresolved clinical problem of BrS (10). Risk factors such as the Tpeak–Tend interval, short-coupled PVC, J wave amplitude, QRS fragmentation, and family history have been identified, but only symptoms and a spontaneous type 1 ECG have consistently been associated with increased ventricular tachycardia/fibrillation risk and have prognostic impacts. In terms of family screening, first-degree relatives with type 1 ECGs should be treated as having BrS. It is recommended that first-degree relatives with normal ECGs have annual ECG follow-ups. Therefore, annual ECG follow-ups might be recommended for the first-degree relatives of the index patient. However, the brother's ECG indicated a conduction delay, which could contribute to BrS in terms of the depolarization hypothesis, and special observation would be considered for him. Family members who carry BrS-associated variants but remain asymptomatic and have normal ECGs indicate that genetic studies are of limited usefulness for determining risk (28). In contrast, even though family gene variants are not determined, there is no reason to exclude BrS if the patients exhibit the Brugada ECG pattern. Therefore, although *SCN5A* is the only undisputed gene thought to cause BrS, genetic testing alone is insufficient to diagnose BrS because variants in this gene can result in various phenotypes. Guidelines do not recommend diagnosis of BrS based on genetics alone (2, 13, 29).

A comprehensive evaluation, including history-taking, ECGs, and genetics could identify family members at risk for BrS. We can apply the Shanghai score system to evaluate members having with suspected Brugada-like ECGs. According to the current guidelines, the results of genetic screening do not influence the prognosis or therapy of the index patient. However, Nishii et al. reported that SCN5A mutation is associated with early and frequent recurrence of ventricular fibrillation in patients with Brugada syndrome (30). Reports on the correlation bewteen genetics and clinical manifestations are ongoing. As in the present family, when genetic incomplete penetrance is suspected, genetic studies are helpful in the search for BrS phenotypes and risk stratification to prevent sudden cardiac death in the family or to alert the family members of this possibility at least.

This case report provides evidence or direction for evaluating ECG phenotypes in a BrS family group. Along with ECG, genetic studies could be considered as an effective method for evaluating families of BrS probands, resulting in optimal management.
